# CD8+ T Cell Migration to the Skin Requires CD4+ Help in a Murine Model of Contact Hypersensitivity

**DOI:** 10.1371/journal.pone.0041038

**Published:** 2012-08-20

**Authors:** Nanna Fyhrquist, Henrik Wolff, Antti Lauerma, Harri Alenius

**Affiliations:** 1 Unit of Excellence for Immunotoxicology, Finnish Institute of Occupational Health, Helsinki, Finland; 2 Team for Biological Mechanisms and Prevention of Work-related Diseases, Finnish Institute of Occupational Health, Helsinki, Finland; 3 Department of Pathology, Helsinki University Central Hospital, Finland; 4 Control of Hypersensitivity Diseases, Finnish Institute of Occupational Health, Helsinki, Finland; 5 Department of Dermatology, Venereology and Allergology, University of Helsinki, Finland; University of Toronto, Canada

## Abstract

The relative roles of CD4+ and CD8+ T cells in contact hypersensitivity responses have not been fully solved, and remain an important question. Using an adoptive transfer model, we investigated the role of the respective T cell subset. Magnetic bead separated CD4+ and CD8+ T cells from oxazolone sensitized C57BL/6 mice were transferred into RAG−/− mice, followed by hapten challenge and analysis of inflammatory parameters at 24 hours post exposure. The CD4+ T cell recipient mice developed partial contact hypersensitivity responses to oxazolone. CD8+ T cells caused significant amplification of the response in recipients of both CD4+ and CD8+ T cells including ear swelling, type 1 inflammatory mediators, and cell killing. Unexpectedly, CD8+ T cells were not sufficient to mediate contact hypersensitivity, although abundantly present in the lymph nodes in the CD8+ T cell reconstituted mice. There were no signs of inflammation at the site of hapten exposure, indicating impaired recruitment of CD8+ T cells in the absence of CD4+ T cells. These data show that CD4+ T cells mediate contact hypersensitivity to oxazolone, but CD8+ T cells contribute with the most potent effector mechanisms. Moreover, our results suggest that CD4+ T cell function is required for the mobilization of CD8+ effector T cells to the site of hapten exposure. The results shed new light on the relative importance of CD4+ and CD8+ T cells during the effector phase of contact hypersensitivity.

## Introduction

Contact hypersensitivity (CHS) is a T cell-mediated immune response to hapten bound to protein carriers in the skin. CHS is referred to as allergic contact dermatitis (ACD) in the clinic, being a common inflammatory skin disease in industrialized countries. ACD is one of the most common occupational diseases, causing a great socioeconomic burden to the society [1].

CD4+ and CD8+ T cells play distinct roles in the expression of CHS responses, however, their exact nature and their modes of action have not been fully resolved, and remain important questions. CD8+ T cells probably provide the most relevant effector mechanisms causing tissue damage, while CD4+ T cells appear multifunctional, involving both effector and regulatory roles [2], [3], [4].

Chemical haptens induce inflammation in the skin and secretion of TNF-alpha and IL-1beta, leading to local vascular activation, and recruitment of T cells. The activation of antigen-specific T cells leads to release of IFN-gamma and TNF-alpha, as well as IL-4 and IL-17, which further stimulate keratinocytes and other local cells in the skin [4]. This promotes upregulation of adhesion molecules and chemokines including CXCL9 and CXCL10, which facilitates the trafficking of T helper 1 (Th1) cells and CD8+ T cells [5].

To study CD4+ and CD8+ effector T cell interaction during the CHS response, we set up a mouse model of adoptive transfer of CHS, in which CD4+ and CD8+ T cells were primed in wild type (WT) mice, isolated and transferred into RAG−/− recipient mice, which were challenged by allergen.

## Materials and Methods

### Animals

C57BL6 and B6.129S7-Rag1^tm1Mom^/J (RAG−/−) mice (Jackson Laboratory) were maintained under pathogen-free conditions. All animal studies were performed under the approval of the local animal ethics committee (Approval number ESLH-2008-07152/Ym-23, Eläinkoelautakunta -ELLA, Etelä-Suomen lääninhallitus).

### Animal treatment protocol

**Figure 1:**
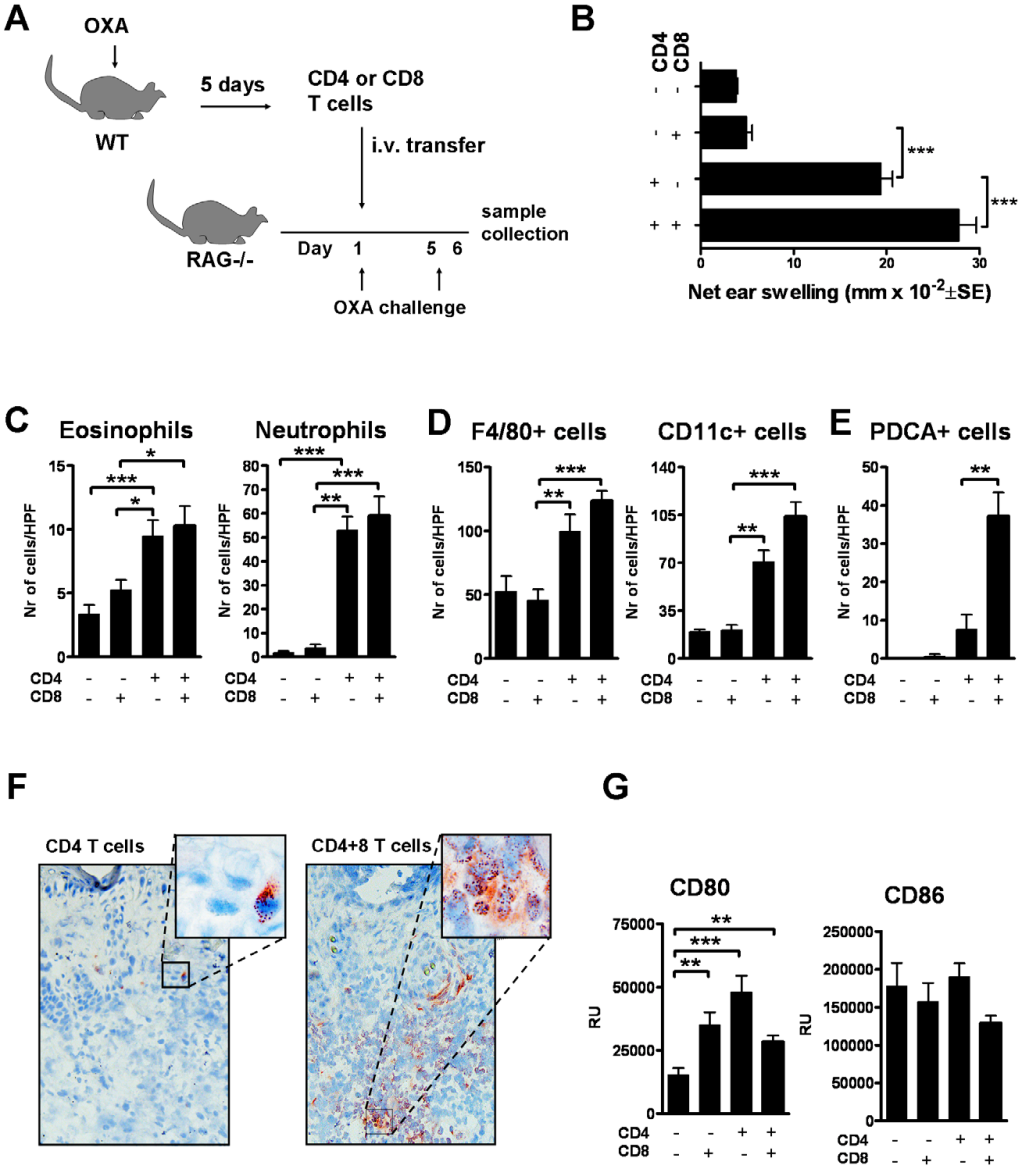
CD4+ T cells mediate ear swelling responses to oxazolone in the RAG−/− model. (A) The CHS adoptive transfer protocol, (B) ear swelling in the T cell recipients, and (C) recruitment of eosinophils and neutrophils, (D) F/80+ and CD11c+ cells, and (E) PDCA+ plasmacytoid dendritic cells to the areas of hapten exposure. (F) Representative immunohistochemical staining of PDCA+ in CD4+ recipients and in mice that received both subsets of T cells. Bars represent mean +SEM (n = 8 mice/group). *, p<0.05, **, p<0.01; and ***, p<0.001.

Female C57BL6 mice were topically treated with oxazolone (50 µl, 10 mg ml^−1^) on shaved backs. Five days later, CD4+ and CD8+ T cells were positively selected by magnetic bead separation (Miltenyi Biotec) from spleens and draining lymph nodes (dLNs), and intravenously (i.v.) transferred (2×10^6^ cells/100 µl PBS/mouse) into RAG−/− mice. T cell recipients were challenged with oxazolone (25 µl, 3 mg ml^−1^) as in Figure 1A to allow for sufficient expansion of antigen-specific T cells, and ear swelling was measured and samples were collected at 24 hours after the last challenge. All experiments were repeated 4 times, with 8 mice per group. Mice receiving PBS i.v. were included in the study.

### Skin histology

Paraffin-embedded ear tissue sections were stained with H&E, for counting eosinophils and neutrophils. Frozen ear tissue sections were stained with anti-CD3+, anti-CD4+, anti-CD8+, and anti-CD11c+ (BD Pharmingen), PDCA+ (Miltenyi biotec), or anti-F4/80+ (Acris antibodies) using the immunoperoxidase staining method. Activated caspase-3 was stained on paraffin-embedded sections using antibody from Cell Signaling Technology, Inc. Stained cells were counted per HPF at ×1000 magnification.

### Measurements of mRNA expression

RNA was extracted from frozen skin and draining lymph node (dLN) samples according to TRIzol instructions ((Invitrogen Life Technologies), followed by reverse transcription and analysis by Quantitative real-time PCR (TaqMan, AbiPrism 7700 Sequence Detector System, Applied Biosystems). Primers and target-specific probes were purchased as pre-developed reagents (Applied Biosystems). Endogenous 18S rRNA was used to normalize gene expression between samples.

### T cell analyses by flow cytometry

Single cell suspensions prepared from dLN and ear tissue samples were stained with anti-CD4 Alexa Fluor 780 (Rat IgG2a, kappa), anti-CD8 Alexa 700 (Rat IgG2a, kappa), anti-CD3 PerCp (Armenian hamster IgG1, kappa) and anti-OX40 PE (Rat IgG1, kappa) antibodies (eBioscience), followed by flow cytometric analysis (BD Pharmingen FACS Canto II).

For IFN-gamma measurements, 1×10^6^ cells per well were incubated with PMA (20 ng/ml) and ionomycine (1000 ng/ml) in complete RPMI 1640 medium (Invitrogen Life Technologies) for 4 hours, with the addition of brefeldin (10 µg/ml) during the last hour of incubation, and stained intracellularly with anti-IFN-gamma (eBioscience).

### Statistical analysis

Student's t-test was used to analyze the data. The data are expressed as mean values ± SEM, n = 8. * p<0.05, ** p<0.01; and *** p<0.001.

## Results

### CD4+ T cells alone but not CD8+ T cells mediate ear swelling responses to oxazolone in the RAG−/− model

To study the roles of CD4+ and CD8+ T cells in CHS, we injected RAG−/− mice i.v. with CD4+ or CD8+ T cells from oxazolone sensitized C57BL donors, followed by topical treatment of the T cell recipients with oxazolone (Figure 1A). The purities of the injected T cell subsets were ∼98%, and the groups that were reconstituted with both CD4+ and CD8+ T cells, received them at a 1∶1 ratio (Figure S1).

CD4+ T cells alone induced a significant ear swelling response to oxazolone at 24 hours post challenge (Figure 1B), and CD4+ and CD8+ T cells acting together induced further swelling of the ears. Surprisingly, CD8+ T cells alone did not induce notable swelling of the ears. However, a small addition of CD4+ T cells (∼10%) to the CD8+ T cell injections resulted in a significant CHS response (Figure S2A). Eosinophils and neutrophils accumulated at the site of hapten exposure in the CD4+ as well as the CD4+ and CD8+ T cell recipients, but were absent in the CD8+ T cell recipients (Figure 1C).

### T cells influence the recruitment and phenotype of antigen presenting cells

Hapten exposure leads to modifications of the DC population in the skin [2]. At 24 hours post challenge, the presence of T cells led to clearly amplified numbers of F4/80+ macrophages and Langerhans cells, as well as CD11c+ DCs at the site of hapten exposure (Figure 1D). Interestingly, the presence of CD8+ T cells was essential to the recruitment of PDCA+ plasmacytoid DCs (pDC): pDCs were low in number in all groups of mice, except in the CD4+ and CD8+ T cell recipients (Figure 1E,F). In the dLNs the expression of co-stimulatory molecule CD80 at the mRNA level, but not CD86, was further induced by the presence of any subtype of T cells (Figure 1G).

### CD8+ T cells fail to reach the skin in the absence of CD4+ T cells

**Figure 2:**
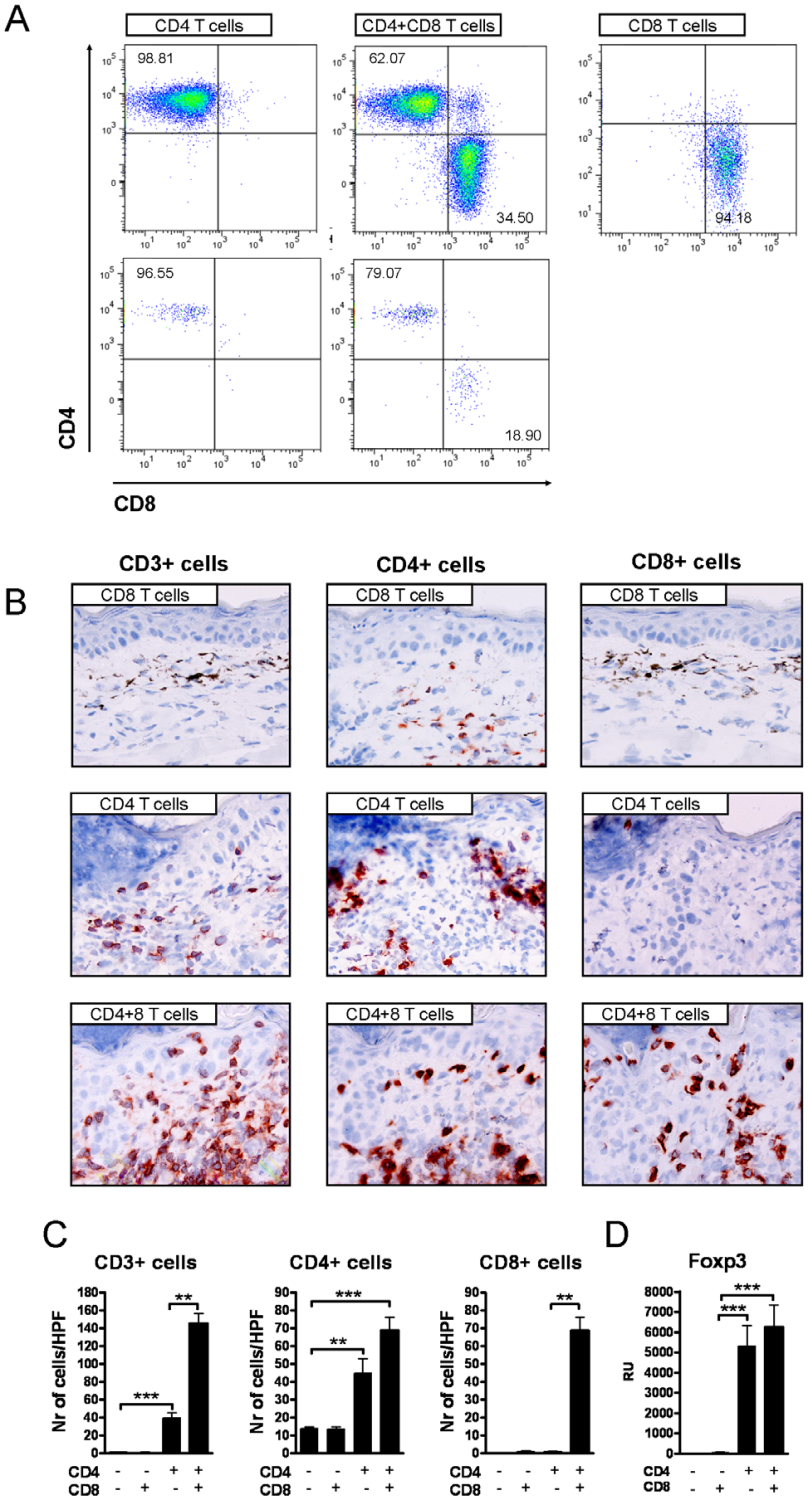
CD3+ cells were absent from the skin in the CD8+ T cell recipients. (A) Flow cytometric analyses of CD4+ and CD8+ T cells in dLN cell suspensions (upper panels) and ear tissue cell supsensions (lower panels). (B) Immunohistochemical staining of CD3+, CD4+ and CD8+ cells in hapten exposed areas, (C) enumeration of the cells and D) Foxp3 expression at the mRNA level. The types of cell transfers are indicated in the insets in the figures. Bars represent mean +SEM (n = 8 mice/group). *, p<0.05, **, p<0.01; and ***, p<0.001.

After topical hapten exposure hapten specific T cells rapidly infiltrate the skin. The purity of the injected T cell subsets remained high (94–98%) throughout the exposure protocol in the dLNs in the RAG−/− mice (Figure 2A, upper panels). In recipients of both subsets, a relatively larger portion of CD4+ T cells infiltrated the skin compared with the dLNs; CD4/CD8 ratios were generally higher in the skin (Figure 2A, two lower panels, Figure S2B,C).

**Figure 3:**
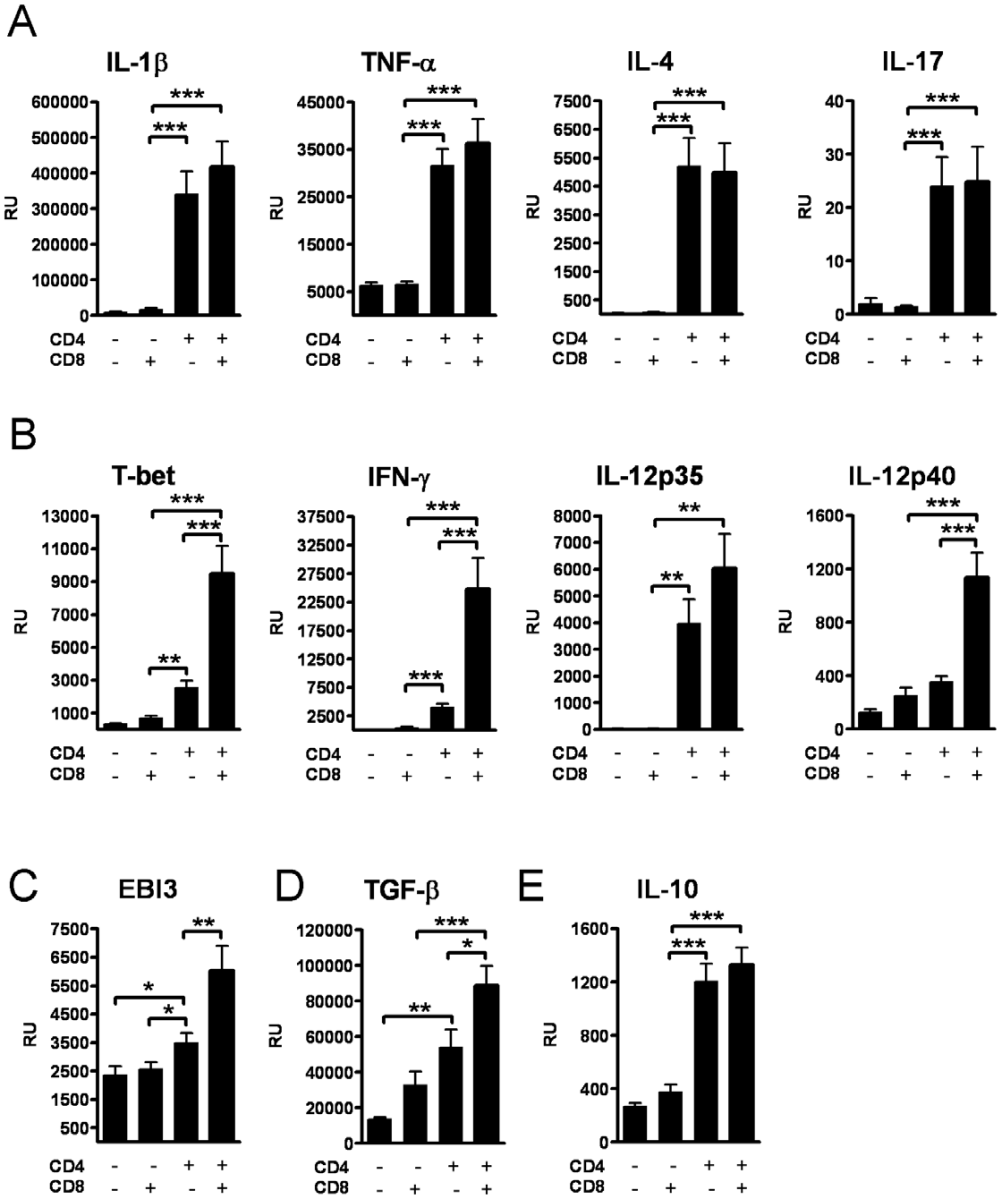
CD8+ T cells amplify Th1 type responses at the site of hapten exposure. Oxazolone induced expression of (A) pro-inflammatory and type 2 mediators, (B,C) type 1 mediators, and (D) TGF-β, and (E) anti-inflammatory IL-10 at the mRNA level in the hapten exposed areas. Bars represent mean +SEM (n = 8 mice/group). *, p<0.05, **, p<0.01; and ***, p<0.001.

Immunohistochemical analyses revealed significant numbers of CD4+ and CD3+ cells, but no CD8+ cells, in the hapten exposed areas in the CD4+ T cell recipients (Figure 2B,C). In the mice that received both CD4+ and CD8+ T cells, CD3+ cell numbers were further increased, and CD8+ cells were detected. By contrast, CD3+ and CD8+ cells were lacking in the skin in the CD8+ T cell recipients, although CD8+ T cells were highly abundant in the dLNs (Figure S3A,B). No CD3+ cells were detected in the RAG−/− mice injected with only PBS (Figure S3C,D). Finally, T regulatory cell marker Foxp3 was present at the mRNA level in the skin in the CD4+ T cell recipients, but not in the other groups of mice (Figure 3F).

### CD8+ T cells contribute potently to the expression of type 1 inflammatory mediators during the CHS response

The CHS response was strong with regards to the local expression of cytokines IL-1beta, TNF-alpha, IL-17 and IL-4 at the mRNA level (Figure 3A) in the CD4+ T cell recipients, with no effects on this part of the response by CD8+ T cells. However, the expression of type 1 inflammatory mediators IFN-gamma, T-bet, IL-12 (p35 and p40) (Figure 3B) and IL-27 (EBI3) (Figure 3C), and multifunctional cytokine TGF-beta (Figure 3D) was significantly weaker when CD8+ T cells were lacking. CD8+ T cells had no effect on the expression of IL-10 (Figure 3E).

**Figure 4:**
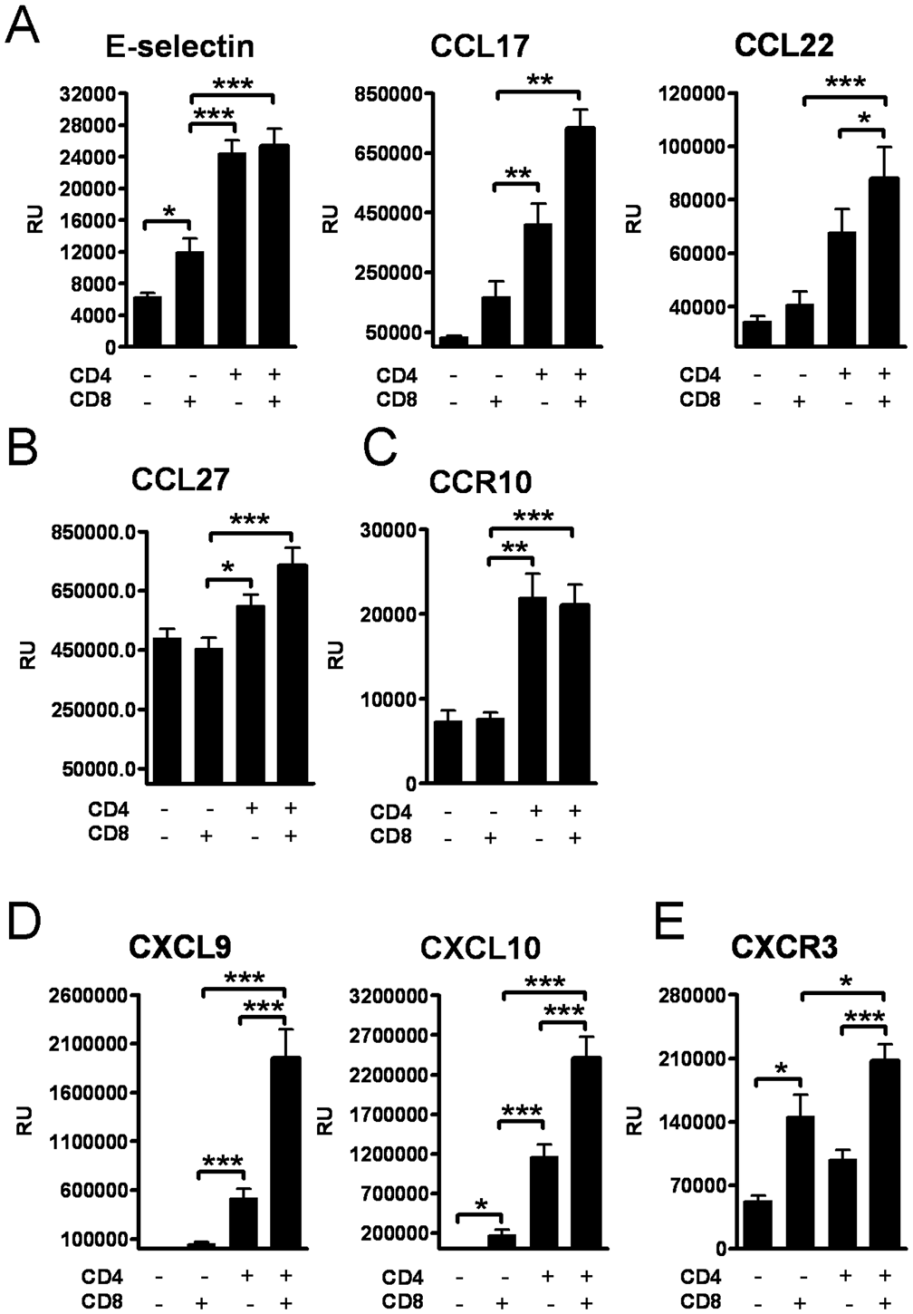
CD8+ T cells amplify the expression of type 1 chemokines. The expression of chemoattractants at the mRNA level: (A) E-selectin, CCL17, CCL22 and (B) CCL27 expression in ear tissue, and (C) CCR10 expression in LN tissue. (D) CXCL9 and CXCL10 expression in ear tissue and (E) CXCR3 expression in LN tissue. Bars represent mean +SEM (n = 8 mice/group). *, p<0.05, **, p<0.01; and ***, p<0.001.

E-selectin, which is involved in the adhesion of leukocytes to the endothelium at the site of hapten exposure, was induced locally in all T cell recipients. Chemokines CCL17, CCL22 and CCL27 which are involved in attracting T-cells specifically to the skin, were induced only in the groups that received CD4+ T cells, or CD4+ and CD8+ T cells (Figure 4A,B). CCL27 is recognized by CCR10+ skin seeking CD4+ T cells [6], and the level of CCR10 mRNA in the dLNs was higher in the CD4+ T cell recipients compared with the CD8+ T cell recipients (Figure 4C), supporting previous findings that primarily CD4+ T cells express CCR10.

The expression of CXCL9 and CXCL10, which are instrumental to the recruitment of Th1 type cells during the CHS response, was significantly lower in the CD4+ T cell recipients which lacked CD8+ T cells (Figure 4D), compared with the group that received both subsets of T cells. In the CD8+ T cell recipients that lacked CD4+ T cells, the expression of CXCL9 and CXCL10 stayed at a basal level post challenge, but their receptor CXCR3 was significantly expressed at the mRNA level in the dLNs in the CD8+ T cell recipients (Figure 4E).

### The numbers activated T cells in the dLNs was similar in all T cells recipients

**Figure 5:**
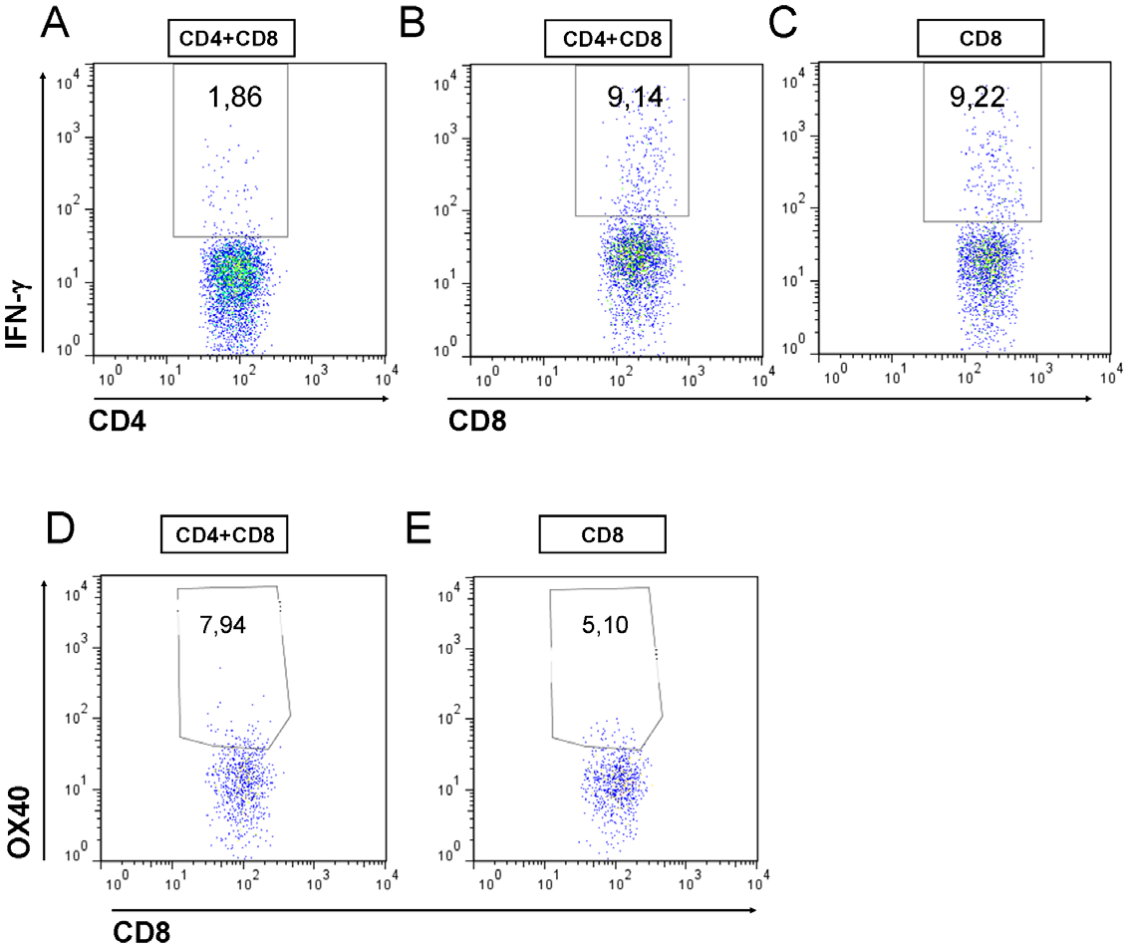
IFN-γ is expressed at normal levels in the T cells in the CD8+ T cell recipients. (A) The share of IFN-γ expressing (A) CD4+ T cells and (B) CD8+ T cells in the dLNs in the recipents of CD4+ and CD8+ T cells, and (C) in CD8+ T cells in the recipients of CD8+ T cells. The share of OX40+ CD8+ T cells in (A) recipients of both CD4+ and CD8+ T cells, and in (B) recipients of CD8+ T cells.

IFN-gamma is a central effector cytokine in CHS responses, produced by activated T cells. In the recipients of both CD4+ and CD8+ T cells, a smaller percentage of CD4+ T cells (Figure 5A) than CD8+ T cells (Figure 5B) produced IFN-gamma, in line with previous studies [7]. In the mice that received either CD4+ T cells or CD8+ T cells, the share of IFN-gamma producing CD4+ (data not shown) or CD8+ T cells (Figure 5C) was at a similar level.

OX40-OX40 ligand interactions mediate co-stimulatory signals between activated T cells and DCs [8]. Activation marker OX40 was expressed at similar levels (5–7%) by CD8+ T cells in the dLNs in the recipients of either CD4+ and CD8+ T cells, or CD8+ T cells only (Figure 5D,E).

### Caspase-3 activation in the epidermis

**Figure 6:**
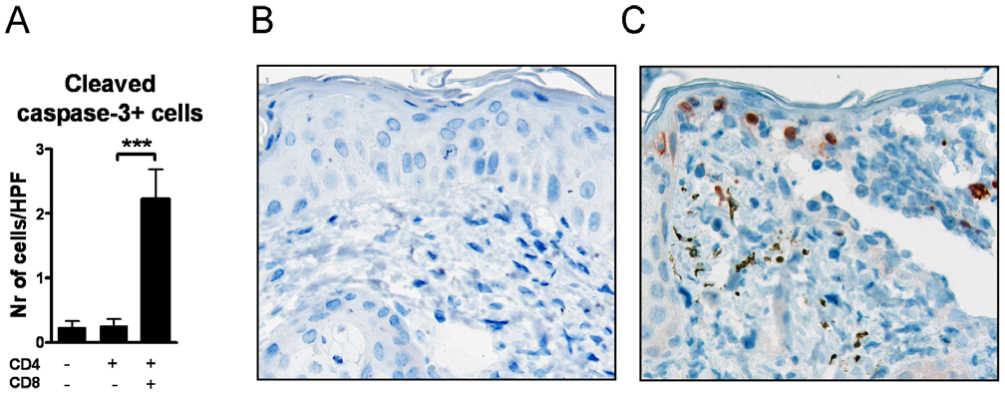
CD8+ T cells induce activation of Caspase-3. (A) Enumeration of cells positive for activated caspase-3 and representative immunohistochemical staining of hapten exposed tissue in (B) CD4+ T cell recipients and in (C) recipients of both T cell subsets.

Keratinocyte apoptosis is a main feature of the CHS response, mediated by cytotoxic activities of mainly CD8+ T cells [2]. Caspase-3 is a key mediator of apoptosis, and its activation requires proteolytic processing of its inactive zymogen into activated p17 and p12 fragments [9]. By using an antibody that detects p17, we observed significant activation of caspase-3 in the epidermal layer of the hapten exposed areas in the recipients of both subsets of T cells. In contrast, in the mice that received CD4+ T cells only, the level of activated caspase-3 was insignificant (Figure 6A, B, C).

## Discussion

Chemical haptens efficiently activate both CD4+ and CD8+ T cells, but according to a number of studies, CD8+ T cells usually dominate the CHS effector response, while CD4+ T cells have a regulatory role [2], [3], [4], [10], [11]. The respective contribution of CD4+ and CD8+ T cells in CHS has been extensively studied using different strategies, including *in vivo* depletion of normal mice with anti-CD4 and anti-CD8 mAbs, transfer of CD4+ or CD8+ T cells to immune incompetent mice, or the use of transgenic mice. Using *in vivo* depletion, Gocinski and Tigelaar [3] were the first to suggest that CD8+ T cells mediate CHS responses. Subsequent experiments in CD8+ T cell deficient MHC class I knock-out mice confirmed a strong effector role for CD8+ T cells, and importantly showed that CD8+ effectors can develop in the absence of CD4+ help [11]. In striking contrast to the established view, our results suggest that CD4+ T cells play an important effector role during the CHS response, acting to a certain extent as effector cells themselves, in addition to guiding CD8+ T cells to the site of allergen exposure.

CD4+ T cells mediated an attenuated CHS response to oxazolone in the RAG−/− model compared with the response induced by CD4+ and CD8+ T cells together. The presence of CD8+ T cell significantly amplified oedema formation (ie. ear swelling), the expression of type 1 inflammatory mediators, cell killing, and the recruitment of PDCA+ cells to the hapten exposed areas. PDCA+ pDCs are known to accumulate in skin lesions in allergic contact dermatitis (ACD), and co-localize with natural killer (NK) cells [12] which they activate through the secretion of cytokines. Moreover, pDCs affect the function and survival of T cells, and promote differentiation, maturation and immunostimulatory functions of DC [13]. Unexpectedly, CD8+ T cells alone were not sufficient to mediate CHS responses to oxazolone in the RAG−/− mice. Although CD8+ T cells were abundant in the dLNs in the CD8+ T cell recipients and expressed normal levels of activation markers (IFN-gamma and OX40), they were not present in the skin at the site of challenge. However, when transferred together with CD4+ T cells, CD8+ T cells efficiently migrated to the skin, and potently amplified the inflammatory response and induced local cell death. Based on these results, we suggest that although CD8+ T cells certainly are important mediators of CHS, they nevertheless require the presence of CD4+ T cells. In previous studies using eg. *in vivo* depletion of cell subsets [3] or MHC class I−/− mice [11], even a minor population of CD4+ T cells might have played an important role early in the immune response and helped CD8+ T cells migrate to the skin. In other disease models, such as viral infection where CD8+ T cells play an important effector role, CD4+ T cells contribute essentially to the mobilization of CD8+ T cells to the site of infection through secretion of IFN-gamma and the induction of local chemokine secretion in the infected tissue [14]. Similarly, ‘pioneering’ CD4+ T cells are required for the entry of pathogenic T cells in the central nervous system [15], advocating for an essential role for CD4+ T cells at the stages after CTL differentiation. Indeed, we observed minor or no induction at all of local chemokines in the RAG−/− mice at the site of hapten exposure when CD4+ T cells were lacking. Further, CCR10, which together with ligand CCL27 is instrumental in guiding T-cells to skin [6], was expressed at a low level in the dLNs in the absence of CD4+ T cells. Our findings will need further studies including comparisons with other contact sensitizers (eg. TNCB or DNCB) and would benefit from confirmation in alternative immune incompetent mouse models. The effects of transferring CD4+ T cells, CD8+ T cells, or CD4+ and CD8+ T cell to the RAG−/− mice in the CHS model are summarized in Table S1.

Contact allergens activate DCs through innate immunity signaling via pattern recognition receptors [16], leading to amplified expression of inflammatory mediators and molecules related to antigen presentation and co-stimulation. In addition, T cells shape the innate immune response through T cell-DC crosstalk and can alter the expression of MHC molecules and co-stimulatory molecules [17]. We observed that the presence of T cells led to the up-regulation of CD80 mRNA, but not CD86 in the dLNs at 24 hours post challenge.

In conclusion, CD4+ T cells mediated partial CHS responses to oxazolone in the absence of CD8+ T cells in a RAG−/− host environment. Unexpectedly, CD8+ T cells were not sufficient to mediate CHS responses when transferred alone, although they were abundantly present in the dLNs in the T cell recipient mice. Adding CD4+ T cells to CD8+ T cells in the cell transfers resulted in full scale CHS responses, suggesting on one hand that CD8+ T cells provide the most relevant effector mechanisms, and on the other hand that CD4+ T cells are required for the mobilization of CD8+ T cells to the skin. The results have implications for future therapeutic approaches, such as targeting specific subsets of T cell populations.

## Supporting Information

Figure S1 **Purity and composition of T cell subsets that were transferred from C57BL6/J into RAG−/− mice:** (A) CD4+ T cells, (B) a 1∶1 cocktail of CD4+ and CD8+ T cells, and (C) CD8+ T cells.
(TIF)

Figure S2 **CD4+ T cells guide CD8+ T cells to the site of allergen exposure.** (A) Injecting RAG−/− mice with 2×10^6^ T cells, at a ratio of 90% CD8+ T cells to 10% CD4+ T cells before hapten challenge, resulted in significant ear swelling. According to flow cytometric analyses, CD4/CD8 ratios were relatively lower in (B) the dLNs compared with (C) the skin.
(TIF)

Figure S3 **Abundant CD8+ cells in the dLNs in CD8+ T cell reconstituted RAG−/− mice.** Immunohistochemical staining of (A) CD3+ cells and (B) CD8+ cells in the dLNs in CD8+ T cell recipient RAG−/− mice. In the RAG−/− mice that received PBS only via the tail vein, there were no CD3+ cells in (C) the dLNs and (D) the hapten exposed ear tissue.
(TIF)

Table S1 **Summary of analysed inflammatory parameters in hapten exposed areas in the RAG−/− mice that received either CD4+ T cells, CD8+ T cells, or CD4+ and CD8+ T cells prior to hapten exposure.**
(TIF)
