# Supplementary figures and images for: CD8+ T Cell Migration to the Skin Requires CD4+ Help in a Murine Model of Contact Hypersensitivity
Source: PLoS One. 2012 Aug 20;7(8):e41038. doi: 10.1371/journal.pone.0041038 (PMC3423415; doi:10.1371/journal.pone.0041038)

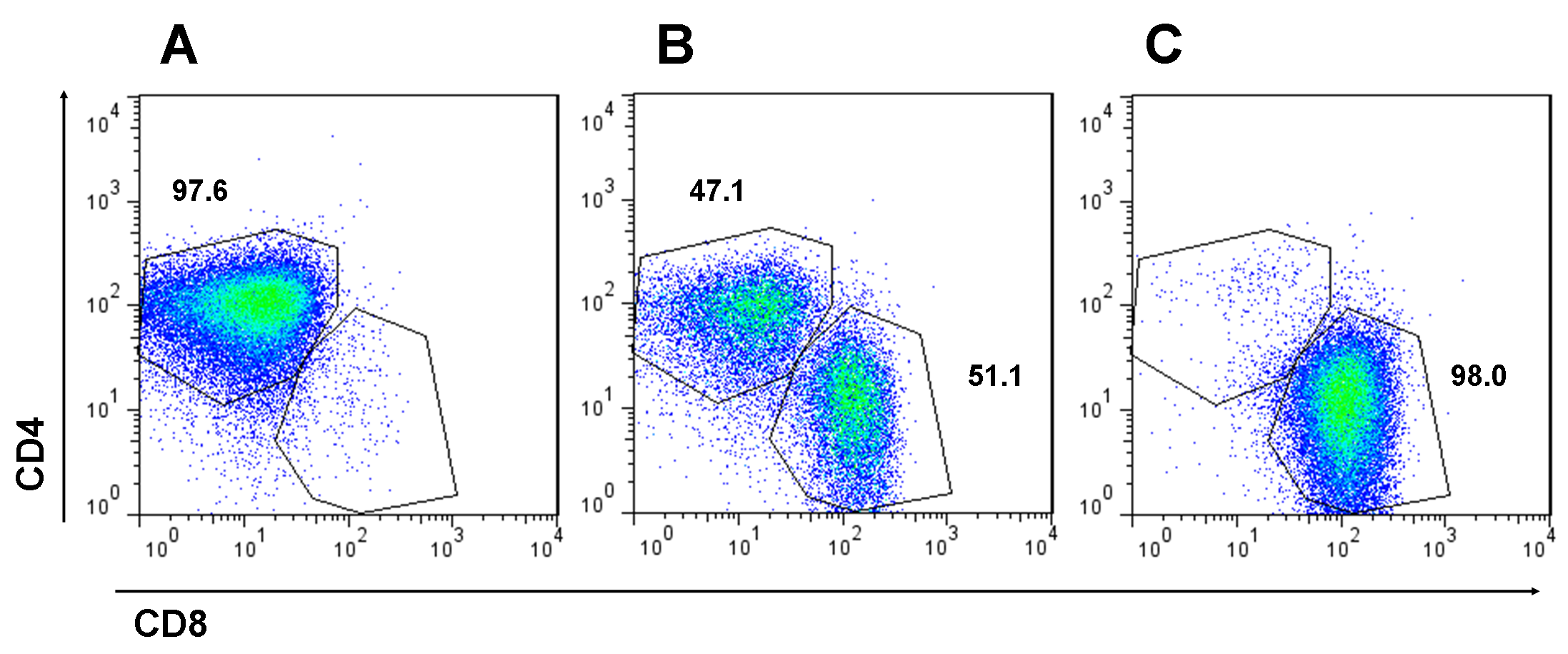

Supplement: Figure S1 — Purity and composition of T cell subsets that were transferred from C57BL6/J into RAG−/− mice: (A) CD4+ T cells, (B) a 1∶1 cocktail of CD4+ and CD8+ T cells, and (C) CD8+ T cells. (TIF) [file pone.0041038.s001.tif]

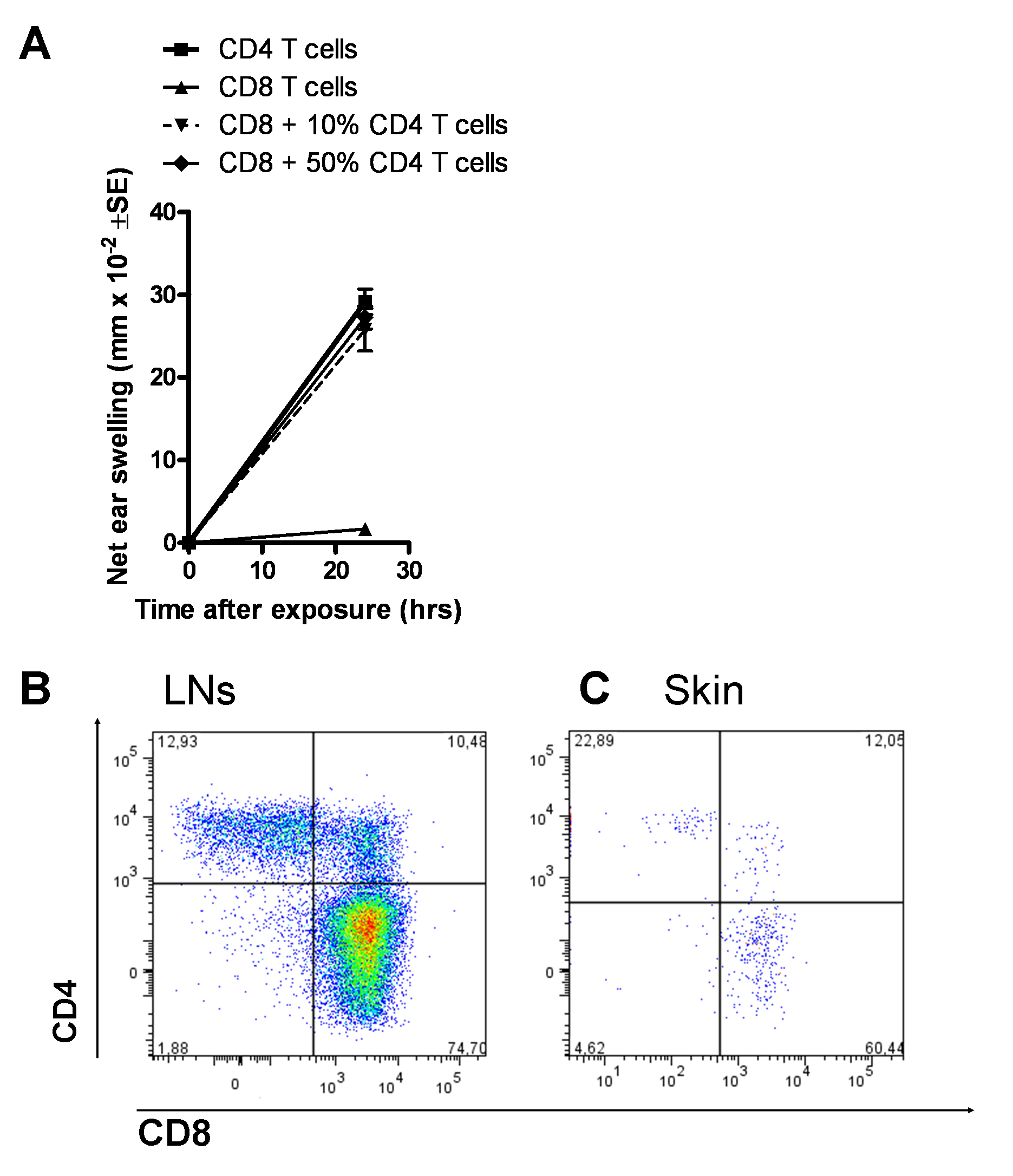

Supplement: Figure S2 — CD4+ T cells guide CD8+ T cells to the site of allergen exposure. (A) Injecting RAG−/− mice with 2×10^6^ T cells, at a ratio of 90% CD8+ T cells to 10% CD4+ T cells before hapten challenge, resulted in significant ear swelling. According to flow cytometric analyses, CD4/CD8 ratios were relatively lower in (B) the dLNs compared with (C) the skin. (TIF) [file pone.0041038.s002.tif]

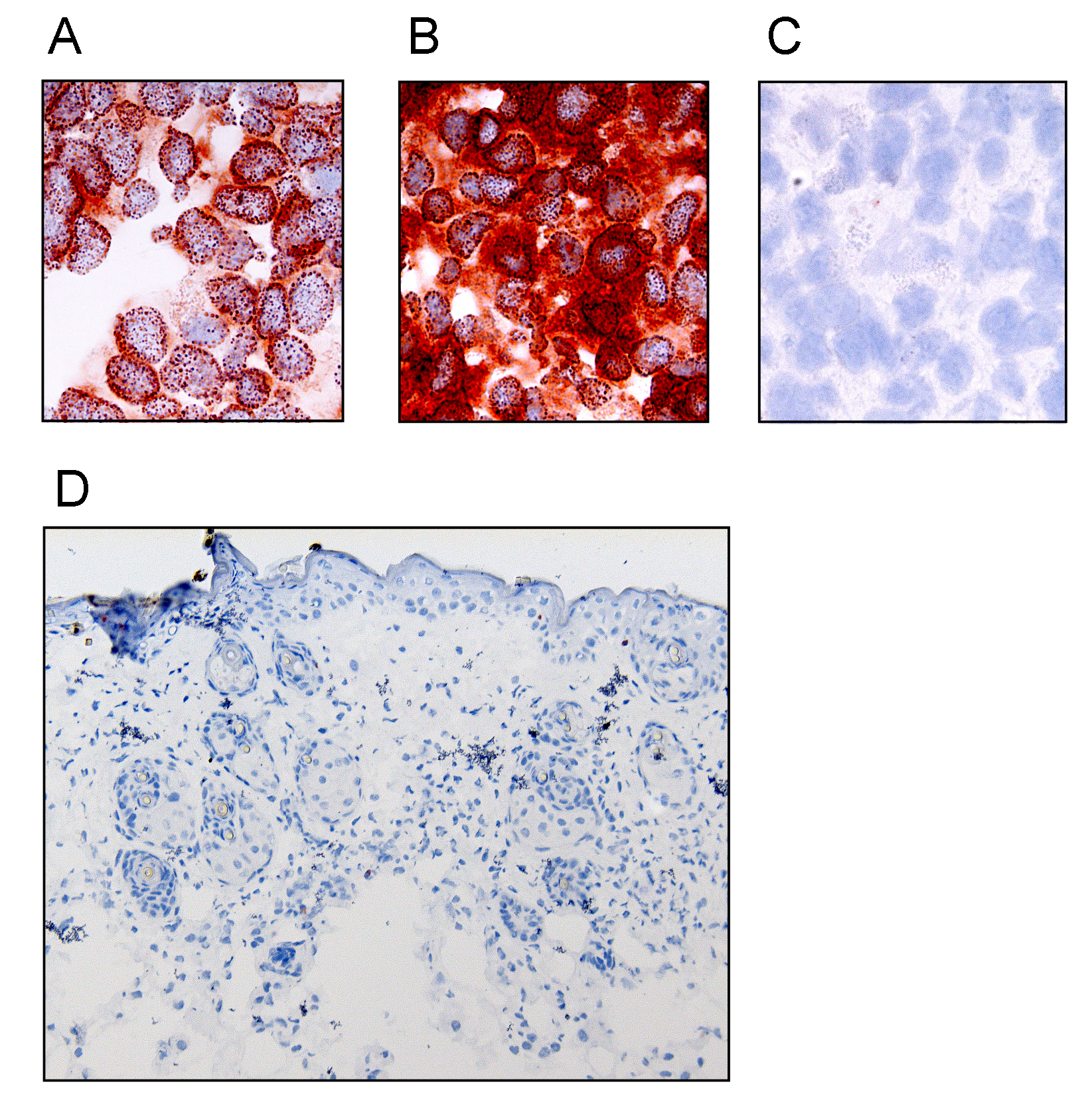

Supplement: Figure S3 — Abundant CD8+ cells in the dLNs in CD8+ T cell reconstituted RAG−/− mice. Immunohistochemical staining of (A) CD3+ cells and (B) CD8+ cells in the dLNs in CD8+ T cell recipient RAG−/− mice. In the RAG−/− mice that received PBS only via the tail vein, there were no CD3+ cells in (C) the dLNs and (D) the hapten exposed ear tissue. (TIF) [file pone.0041038.s003.tif]

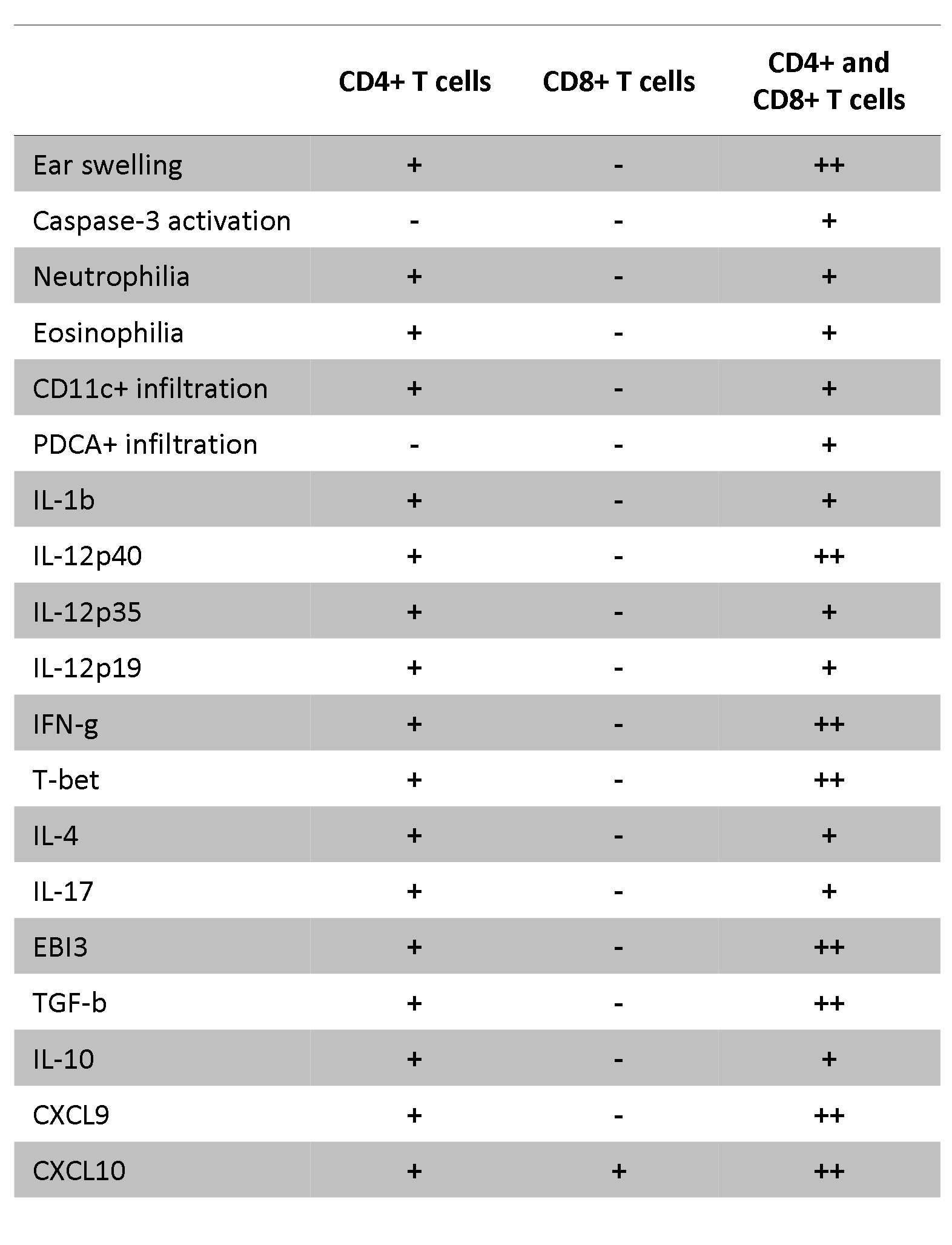

Supplement: Table S1 — Summary of analysed inflammatory parameters in hapten exposed areas in the RAG−/− mice that received either CD4+ T cells, CD8+ T cells, or CD4+ and CD8+ T cells prior to hapten exposure. (TIF) [file pone.0041038.s004.tif]
